# Role of sodium leak channel (NALCN) in sensation and pain: an overview

**DOI:** 10.3389/fphar.2023.1349438

**Published:** 2024-01-11

**Authors:** Donghang Zhang, Yiyong Wei

**Affiliations:** ^1^ Department of Anesthesiology, West China Hospital, Sichuan University, Chengdu, China; ^2^ Department of Anesthesiology, Longgang District Maternity and Child Healthcare Hospital of Shenzhen City (Longgang Maternity and Child Institute of Shantou University Medical College), Shenzhen, China

**Keywords:** NALCN, DRG, spinal cord, pain, sensation

## Abstract

The sodium leak channel (NALCN) is widely expressed in the central nervous system and plays a pivotal role in regulating the resting membrane potential (RMP) by mediating the Na^+^ leak current. NALCN was first reported in 1999, and since then, increasing evidence has provided insights into the structure and functions of NALCN. As an essential component of neuronal background currents, NALCN has been shown to be involved in many important physiological functions, particularly in the respiratory rhythm, as NALCN mutant mice have a severely disrupted respiratory rhythm and die within 24 h of birth. Many patients with NALCN mutations also develop serious clinical syndromes, such as severe hypotonia, speech impairment, and cognitive delay. Recently, emerging studies have clarified the human NALCN structure and revealed additional properties and functions of NALCN. For instance, accumulating evidence highlights that the NALCN is involved in normal sensation and pain. Here, we review the current literature and summarize the role of the NALCN in sensation and pain.

## Introduction

Despite the predominant role of potassium leak conductance in maintaining the resting membrane potential (RMP) of neurons, the RMP of most mammalian neurons is considerably depolarized to the potassium equilibrium potential, suggesting that other conductance coexists (Ren, 2011; Lu and Feng, 2012). The sodium leak channel (NALCN) is widely expressed in neurons of the central nervous system (CNS) and has been confirmed to contribute to the RMP in neurons and control its excitability (Lu et al., 2007; Lutas et al., 2016; Shi et al., 2016; Cobb-Lewis et al., 2023). Growing evidence indicates that NALCN is essential for maintaining many biological functions, such as rhythmic behaviors and locomotor behaviors, in both mammals and invertebrates (Lu et al., 2007; Xie et al., 2013; Gao et al., 2015; Yeh et al., 2017; Zhou et al., 2022). Moreover, an increasing number of patients with NALCN mutations have been reported to have severe manifestations similar to those in animals (Lozic et al., 2016; Angius et al., 2018; Bourque et al., 2018; Campbell et al., 2018; Karimi et al., 2020). Therefore, NALCN is essential for maintaining vital functions in organisms.

**FIGURE 1:**
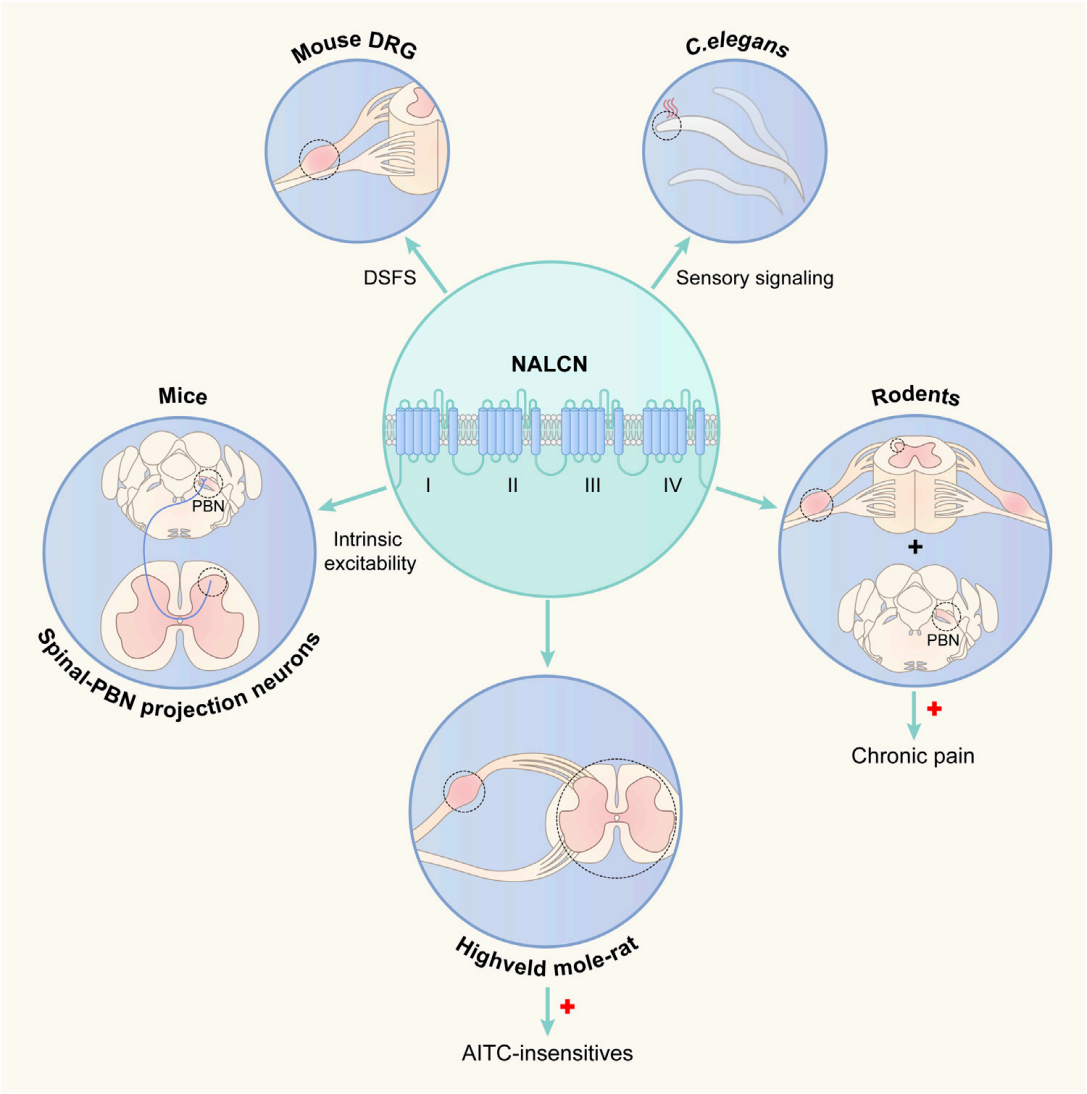
Schematic plot of the association between the NALCN and sensation. NALCN: sodium leak channel; DRG: dorsal root ganglion; DSFs: depolarizing spontaneous fluctuations of membrane potential; PBN: parabrachial nucleus; AITC: allyl isothiocyanate. 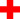
means increased NALCN expression; 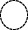
means study regions.

NALCN was first detected in the rat brain in 1999 (Lee et al., 1999). Since then, NALCN has been found widely expressed in almost all neurons of the CNS in both mammals and invertebrates (Lu et al., 2007; Yeh et al., 2008; Ren, 2011; Lu and Feng, 2012). In the mouse brain, NALCN is also present in oligodendrocytes and at a very low level in astrocytes (Cahoy et al., 2008). The expression pattern suggested that NALCN plays fundamental roles. Recent studies have shown that NALCN is also abundant in the spinal cord and dorsal root ganglion (DRG) of rodents (Zhang et al., 2021; Li et al., 2021), indicating that NALCN might be implicated in important animal behaviors, such as pain and sensation. Increasing *in vitro* and/or *in vivo* evidence has shown that NALCN is associated with physiological sensation or pain (Figure 1) (Ford et al., 2018; Eigenbrod et al., 2019; Saro et al., 2020; Zhang et al., 2021; Li et al., 2021; Tian et al., 2023; Wu et al., 2023). Here, we will review the current literature to summarize the contribution of the NALCN to sensation and pain.

## *In vitro* evidence

Ford et al. first showed that NALCN controls the intrinsic excitability of spinal-parabrachial nucleus (PBN) projection neurons in developing mice (Ford et al., 2018). Pharmacological inhibition or knockout of NALCN suppresses the intrinsic excitability of spinal-PBN neurons. Furthermore, the authors demonstrated that substance P (SP) can activate NALCN and enhance excitability in spinal-PBN neurons via Src kinase signaling, which is consistent with findings in the brain from previous studies (Lu et al., 2009; Ren, 2011). Their findings suggest that NALCN conductance in spinal-PBN projection neurons may govern ascending nociceptive transmission to the brain and thereby modulate pain perception. However, this study did not validate the role of NALCN in pain sensation or transduction using *in vivo* experiments. In addition, whether NALCN regulates the intrinsic excitability of spinal neurons in adults is unclear.

Depolarizing spontaneous fluctuations of membrane potential (DSFs) are suggested to control the spontaneous discharge of nociceptors, which is associated with prior pain. Tian et al. showed that NALCN partially contributed to regulating the amplitude and frequency of DSFs in nociceptors using nonspecific inhibitors of NALCN, namely, Gd^3+^ and L-703606 (Tian et al., 2023). Highly selective inhibitors or specific knockdown or knockout of NALCN are needed to determine the role of NALCN in DSFs. In addition to NALCN, their findings also highlight an important contribution from diverse ion channels permeable to Na^+^ and/or Ca^2+^, such as Nav1.7, Nav1.8, Nav1.9, TRPV1, TRPA1, TRPM4, and N-type Ca^2+^ channels, some of which have been confirmed to be associated with pain conditions. Characterization of the relative contributions of these ion channels to the generation of DSFs under pathological conditions may guide the development of more effective molecular targets for the control of pain.

## *In vivo* evidence

Eigenbrod et al. first provided direct evidence that NALCN is associated with pain sensation *in vivo* (Eigenbrod et al., 2019). These authors aimed to identify the evolutive mechanism of pain insensitivity in multiple African rodents. They found that NALCN was significantly upregulated in the dorsal root ganglia and spinal cord of highveld mole-rats, which was suggested to cause insensitivity to allyl isothiocyanate (AITC)-pain. In *in vitro* experiments, overexpression of NALCN channels in cultured cells increased background sodium currents, which led to a decrease in cellular input resistance and depolarized RMP, thereby preventing action potential firing by inactivating voltage-gated sodium channels. Thus, extremely increased expression of NALCN at nociceptor terminals could dampen excitation after TRPA1 activation in highveld mole-rats. Notably, verapamil, a potent blocker of NALCN, could reveal behavioral sensitivity to AITC in highveld mole-rats. However, as verapamil is also a calcium channel antagonist, the exact contribution of NALCN should be determined using completely specific NALCN blockers or by knocking down or knocking out NALCN in the DRG and spinal cord of highveld mole-rats.

Zhang et al. reported that NALCN is also associated with the development of pathological pain in rodents (Zhang et al., 2021). Like in the brain, NALCN was abundantly expressed in the peripheral DRG and spinal cord neurons of rats and mice. In a chronic constriction injury (CCI) model, NALCN expression and function in the DRG and dorsal spinal cord were elevated, which contributed to neuronal sensitization and neuropathic pain, as well as complete Freund’s adjuvant (CFA)-induced inflammatory pain (Li et al., 2021). Interestingly, these findings appear to be contrary to those from the study of Eigenbrod et al. (2019), which may be explained by the differentially elevated levels of NALCN expression. The expression level of NALCN determines the extent of RMP depolarization, thereby leading to pain insensitivity or sensitivity. Significant depolarization of the RMP by extreme overexpression of NALCN can inactivate voltage-gated sodium channels and dampen neuronal excitability in highveld mole-rats (Eigenbrod et al., 2019), while the RMP is depolarized by less than 10 mV, which leads to neuronal sensitization in CCI-induced hyperalgesia (Zhang et al., 2021). Nevertheless, all this evidence points to NALCN as an underlying molecular target for pain sensation.

In addition to the DRG and spinal cord, the NALCN in the brain was also found to be related to the regulation of pain. Wu et al. showed that knocking down NALCN in lateral parabrachial nucleus (PBL) glutamatergic neurons alleviated CFA-induced pain in mice (Wu et al., 2023). Their findings further suggested that the NALCN in PBL glutamatergic neurons regulates inflammatory pain via PBL-central nucleus amygdala (CeA) projections. However, the authors did not use patch recordings or calcium imaging to detect the excitability of PBL glutamatergic neurons when NALCN expression was knocked down, except for simply examining the change in c-fos expression. Moreover, researchers have not confirmed whether NALCN knockdown in PBL induces other abnormal phenotypes.

Saro et al. used *in vivo* experiments to show that NALCN was involved in sensory and thermal signal processing in *C. elegans* (Saro et al., 2020). In their study, two mutated genes, nca-1 and nca-2, were used to examine the role of NALCN in primary nociceptors in *C. elegans*. They showed that both mutations reduced the magnitude of heat-evoked calcium changes and affected thermal sensitivity, while nca-2 mutations also influenced sensory gain and signal kinetics during termination of thermal stimuli. Given the high conservation of NALCN expression and functions across animals, this study may provide new insights into the molecular machinery of ascending nociceptive pathways in sensory perception and important behaviors.

## Strengths and weaknesses of the current evidence regarding the role of NALCN in pain and sensation

Current evidence from both *in vivo* and *in vitro* experiments indicates that NALCN plays a pivotal role in controlling pain (Eigenbrod et al., 2019; Zhang et al., 2021; Li et al., 2021; Wu et al., 2023). A previous study also indicated that the NALCN determines the intrinsic excitability of spinal projection neurons using *in vitro* experiments (Ford et al., 2018), which suggests that the NALCN contributes to sensory conduction and pain perception. However, behavioral tests using NALCN knockdown or knockout techniques are still needed to confirm the role of NALCN in controlling sensory conduction from peripheral sites to the central nervous system. Moreover, the role of NALCN in specific neuronal subtypes of the spinal cord needs to be clarified because of the component heterogeneity in both human (Yadav et al., 2023; Zhang et al., 2023) and mouse spinal neurons (Sathyamurthy et al., 2018; Russ et al., 2021). Additionally, the upstream and downstream molecular targets that mediate the effects of NALCN on pain and sensation have not been identified. Notably, two important subunits of NALCN, namely, UNC80 and UNC79, are essential for normal NALCN function (Ren, 2011; Lu and Feng, 2012). Therefore, it will be interesting to determine the role of UNC80 and UNC79 in pain and sensation, which might also be novel targets for controlling pain. More importantly, although NALCN is also widely expressed in the human DRG and spinal cord (Zhang et al., 2021; Zhang et al., 2022), evidence that NALCN regulates pain in humans has not yet been found. Future studies are needed to explore whether NALCN is a key target for controlling human pain, which will spur drug development and facilitate successful clinical translation from rodent findings.

According to the results of recent studies, the role of the NALCN in pain and sensation in peripheral DRG and spinal cord neurons (Ford et al., 2018; Eigenbrod et al., 2019; Zhang et al., 2021; Li et al., 2021; Tian et al., 2023) appears to be more important than that in brain neurons (Wu et al., 2023). However, NALCN may be associated with central sensitization induced by chronic pain because NALCN is widely expressed in the central nervous system and controls neuronal excitability (Lu et al., 2007; Ren, 2011). Future studies may uncover the contribution of the NALCN in central neurons, especially neuronal subtypes, as well as the involved neural circuits in the regulation of pain perception. For example, one study revealed that the NALCN controls the neuronal excitability of spinal-PBN projection neurons (Ford et al., 2018). Therefore, determining the role of NALCN in the spinal-PBN pathway in pain and physiological sensory signaling will be interesting. However, whether NALCN is an underlying molecular target in pain-related brain nuclei, such as the ventral tegmental area (VTA) (Markovic et al., 2021), the central nucleus of the amygdala (CeA) (Zhou et al., 2019; Lin et al., 2022), and the basal forebrain (Zhou et al., 2023), that regulate pain also remains elusive.

## Perspectives

Although increasing evidence suggests that NALCN may be a promising molecular target for treating pain conditions, one important obstacle is that highly specific blockers for NALCN are unavailable. N-Benzhydryl quinuclidine compounds have been suggested to be promising inhibitors of NALCN with a certain level of selectivity in *in vitro* experiments (Hahn et al., 2020), but their role in pain has not yet been explored. Recently, the structural architecture of the human NALCN has been elucidated (Kschonsak et al., 2020; Xie et al., 2020; Zhou et al., 2022; Kschonsak et al., 2022), which will substantially facilitate the discovery of highly selective drugs to potentially treat NALCN-related disorders, such as pain, by blocking NALCN. Notably, global inhibition of NALCN may cause abnormal functional outcomes, such as respiratory depression; therefore, the discovery of inhibitors targeting the peripheral nervous system, such as the DRG, may be more effective and safer. Therefore, this novel drug should have high selectivity for both NALCN and the peripheral nervous system, which might also be progressed to a clinical therapy. Considering the convenience of the drug delivery route, oral or intravenous administration of NALCN inhibitors is preferred over direct DRG or intrathecal injection, especially when repeated or multiple injections are needed. Notably, whether reduced NALCN activity in the DRG and/or spinal cord leads to abnormal biological functions, such as motor behaviors, needs to be validated in future studies. A recent study revealed that NALCN is also expressed in the glial cells of the DRG and spinal cord in both humans and rodents (Zhang et al., 2022). The role of NALCN in glial cells should also be determined, as the activities of glial cells are involved in pain sensation. However, no clinical patients with NALCN mutations were reported to have sensory or pain disorders; therefore, it will be interesting to test the sensory functions of patients with NALCN mutations.
